# Antioxidant Activities of *Melittis melissophyllum* L. (Lamiaceae)

**DOI:** 10.3390/molecules16043152

**Published:** 2011-04-14

**Authors:** Biljana Kaurinovic, Mira Popovic, Sanja Vlaisavljevic, Milena Raseta

**Affiliations:** Department of Chemistry, Biochemistry and Environmental Protection, Faculty of Science, University of Novi Sad, Trg Dositeja Obradovica 3, 21000 Novi Sad, Serbia

**Keywords:** *Melittis melissophyllum* L. extracts, free radicals, oxidative stress, *in vitro *tests, *in vivo* experiments

## Abstract

Extracts of *Melittis melissophyllum* leaves in ether, chloroform, ethyl acetate, *n*-butanol and water were evaporated to dryness and dissolved in 50% ethanol to make 10% (w/v) solutions. The potential protective action of the extracts was assessed by the corresponding *in vitro *and *in vivo *tests. In the *in vitro *experiments extracts were tested as potential scavengers of free radicals (DPPH, O_2_^·^^−^, NO, and OH radicals), as well as inhibitors of liposomal peroxidation (LPx). The results obtained show that all extracts (exept n-BuOH extract) are good scavengers of radicals and reduce LPx intensity in liposomes, which points to their protective (antioxidant) activity. *In vivo *experiments were concerned with antioxidant systems (activities of GSHPx, GSHR, Px, CAT, XOD, GSH content and intensity of LPx) in liver homogenate and blood-hemolysate of experimental animals after their treatment with extracts of *M. melissophyllum* leaves, or in combination with CCl_4_. On the basis of the results obtained it can be concluded that the examined extracts have protective (antioxidative) effect and this antioxidative behaviour is more pronounced in liver than in blood-hemolysate. The reason is probably the fact that liver contains other enzymatic systems, which can also participate in the antioxidative mechanism. Of all the extracts the H_2_O one showed the highest protective activity.

## 1. Introduction

One of the paradoxes of life on Earth is that, on the one hand, oxygen is necessary for the life of aerobic organisms. On the other hand, increased concentrations of oxygen and especially its reactive metabolites (reactive oxygen species) may lead to the development of numerous diseases. A major source of free radicals in biological systems is molecular oxygen (O_2_). By interacting with fundamental cell structures and biomolecules, reactive oxygen species (ROS) may lead to a number of physiological and patophysiological disorders such as Alzheimer’s, Parkinson’s, ischemia – reperfusion injury, coronary atherosclerosis, diabetes mellitus, hypertension, and cancer genesis, as well in the aging process [1,2]. To prevent and/or treat the damage caused by oxygen free radicals, which initiate the process of peroxidation of the cell lipid membrane, use is made various substances capable of inhibiting free-radical reactions in the cell. 

The changes in the oxidative status or the natural protection system indicate the need for an antioxidative therapy. Active compounds from medicinal herbs (flavonoids, flavolignans, and polyphenols) are natural antioxidants which should be preferably used both for the prevention and antioxidative therapy of various diseases [3]. It is well-known that most spices, especially those belonging to the Lamiaceae family, possess a wide range of biological and pharmacological activities. Since ancient times, they have been used to improve the flavor and the organoleptic properties of different types of food. Furthermore, the use of aromatic plants and spices in phytotherapy is mostly related to different activities of their essential oils, such as antimicrobial, spasmolytic, carminative, hepatoprotective, antiviral, anticarcinogenic, etc. activities [4,5].

*Melittis melissophyllum* L. (Lamiaceae) leaves contain no more than 0.13% of essential oil which is of complex and variable composition. Among the more than 50 compounds identified to date, citronellal (dominantly the (*R*) enantiomer), β-caryophyllene, β-caryophyllene oxide, germacrene-D, nerol, geranial, citronellol, and geraniol amount to about 70% of the oil [6]. The composition is similar to that of lemongrass, but balm oil can be identified by its typical pattern of chiral compounds; for example, almost enantiomerically pure (*R*)-(+)-methyl citronellate is a good indicator of true balm oil. Beside that, leaves contain flavonoids (apigenin, luteolin, and kaempferol), triterpenes, phenolic acids (rosmarinic, caffeic, and chlorogenic acid), sterols and salts [7]. The essential oil exhibits spasmolitic action and acts as a muscle relaxant, sedative, narcotic, antibacterial, and antifungal [8]. 

In view of the above it was of interest to study the potential *in vitro* protective action of various extracts of *M. melissophyllum*, to assess their potential capacity as scavengers of free radicals. Furthermore, extracts of *M. melissophyllum* leaves were used to study their *in vivo *effects on some antioxidant systems in the experimental animal liver and blood-hemolysate in combination with and without carbon tetrachloride (CCl_4_), in order to prove possible antioxidative properties of this, very often used folk medicine remedy (secondary medicaments).

## 2. Results and Discussion

### 2.1. In vitro experiments

Results of determination of total flavonoids in *M. melissophyllum* leaves extracts are given in Table 1. 

**Table 1:** Content of total flavonoids (mg/g) in extracts of leaves of *M. melissophyllum.*

Extract	Et_2_O	CHCl_3_	EtOAc	n-BuOH	H_2_O
**Leaf**	0.79	1.14	1.31	0.41	1.98

The amount of flavonoids in extracts plays an important role in their antioxidative behavior. The richest in flavonoids proved to be the water extract, while the *n*-BuOH extract contains the lowest amount of these active substances. 

Antiradical activity was observed in the study of *M. melissophyllum* leaves extracts in different solvents on the content of DPPH, O_2_^·^^−^ and NO radicals (Table 2), whereby the H_2_O extract exhibited the strongest inhibitory effect, as the IC_50 _value was achieved with the lowest concentration.

In the DPPH assay, the ability of the investigated extracts to act as donors of hydrogen atoms or electrons in transformation of DPPH^•^ into its reduced form DPPH-H was investigated (Table 2). All of the assessed extracts were able to reduce the stable, purple-colored radical DPPH into yellow-colored DPPH-H reaching 50% of reduction with an IC_50_ as follows: 9.21 μg/mL for H_2_O, 11.34 μg/mL for EtOAc, 11.92 μg/mL for CHCl_3_, 17.21 μg/mL for *n*-BuOH, and 18.09 μg/mL for Et_2_O extract. Comparison of the DPPH scavenging activity of the investigated extracts with those expressed by BHT (14.31 μg/mL) showed that H_2_O, EtOAc, and CHCl_3_ extracts expressed stronger antioxidant effects. When investigating neutralization of O_2_^·^^−^ and NO radicals, water extract has also exhibited the greatest ability of their scavenging. 

**Table 2:** IC_50_ values (μg/mL) of the neutralization of DPPH, O_2_^·^^−^ andNO radicals with *M. melissophyllum* extracts.

			IC_50_ (μg/mL)			
Extract	Et_2_O	CHCl_3_	EtOAc	*n*-BuOH	H_2_O	BHT
**DPPH radical**	18.09	11.92	11.34	17.21	9.21	14.31
**O_2_** **^°^** **^−^ radical**	8.11	7.91	14.42	13.26	6.38	10.46
**NO radical**	8.04	7.42	8.91	9.12	6.17	8.63

For the neutralization of DPPH, O_2_^·^^−^ and NO radicals, the most responsible compounds were flavonoids and phenolic acids present in the leaves of *M. melissophyllum* [9,10], so obtained results can be related to the experiments in which the total amount of flavonoids was determined (Table 1.), which show that water extract of *M. melissophyllum* leaf contains the largest amounts of total flavonoids. It is well known that some flavonoids and phenolic acids isolated from *M. melissophyllum* possess certain biological and pharmacological activity [11,12]. For example, apigenin, one of the flavonoids present in *M. melissophyllum*, was shown to express strong antioxidant effects, increasing the activities of antioxidant enzymes and, related to that, decreasing the oxidative damage to tissues [10,13]. Luteolin is also thought to play an important role in the human body as an antioxidant, a free radical scavenger, an agent in the prevention of inflammation, a promoter of carbohydrate metabolism, and an immune system modulator. These characteristics of luteolin are also believed to play an important part in the prevention of cancer. Multiple research experiments describe luteolin as a biochemical agent that can dramatically reduce inflammation and the symptoms of septic shock [14]. Furthermore, it can be supposed that such antiradical activity is caused, besides flavonoids, also by triterpenoids acids (especially ursolic, oleanolic, and pomolic acid) since nonpolar solvents also exhibited high antiradical potential [15,16,17]. 

**Figure 1:**
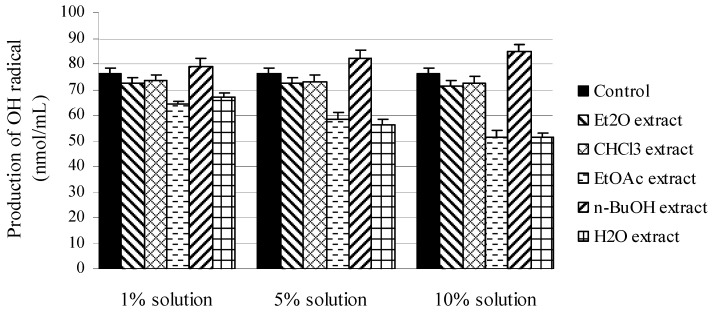
Inhibition of degradation of 2-deoxyribose by different extracts of *M. melissophyllum* in the deoxyribose assay.

The hydroxyl RSC of the examined *M. melissophyllum* extracts (1%, 5% and 10%) measured by the deoxyribose assay is shown(Figure 1) The protective effects of the extracts on 2-deoxy-D-ribose were assessed as their ability to remove hydroxyl radicals, formed in Fenton reaction, from the test solution and to prevent its degradation. *M. melissophyllum* extracts have exhibited different behavior related to production of OH radicals. Ethyl acetate and water extracts at all investigated concentrations expressed OH radical inhibition. A stronger inhibition of OH radical production was expressed by the water extracts, especially the 10% solution (51.2 ± 1.7 nmol/mL) in comparison with the control (76.3 ± 2.1 nmol/mL). 

This behaviour of the water extract can be explained by the presence of phenolic acids and flavonoid glycosides [18]. It is known from the literature [19,20] that bastard balm, and especially its leaves, is characterised by a high content of flavonoid glucosides. Luteolin 7-glucoside has been isolated from the *M. melissophyllum* flower and leaf, whereas of phenolic compounds were detected rosmarinic, caffeic and chlorogenic acid in leaves and ursolic acid in the plant root and leaf [20].

On the basis of this it can be supposed that the very pronounced protective effect of the H_2_O and EtOAc extracts of *M. melissophyllum* is due to the presence of flavonoids and glucosides, namely of luteolin, either being present as free or in the form of its glucosides. The suggested mechanism of flavonoid antioxidative action is as follows: the double bond in position 2,3 is conjugated with the C_4_-carbonyl group, and free OH groups (C_5_, C_3_ and C_7_) can form chelates with ions of d-elements. Once formed, complex with Fe^2+^ ion prevents formation of OH^·^ radicals in Fenton’s reaction [21]. It was determined that rosmarinic acid has stronger antioxidant effect than vitamin E. Rosmarinic acid prevents cell damage caused by free radicals and reduces the risk of cancer and atherosclerosis. In contrast to the histamines, rosmarinic acid prevents activation of the immune system cells that cause swelling and fluid collection [22]. n-BuOH extract has prooxidative effects, when other two extracts (etheric and chloroformic) don`t show neither ani-, neither pro-oxidative effect. 

**Figure 2:**
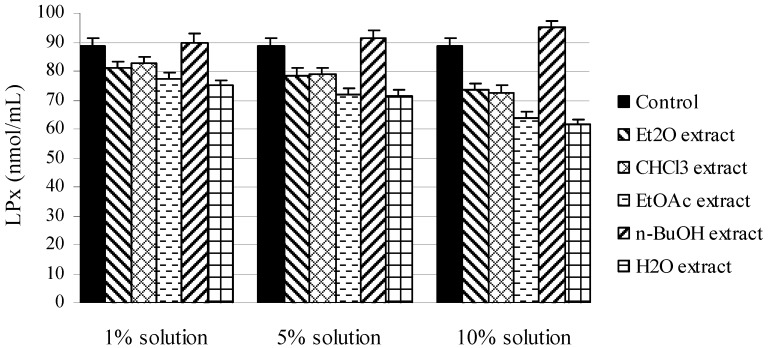
Inhibition of LP in Fe^2+^/ascorbate system of induction by different extracts of *M. melissophyllum* in the TBA assay.

The protective effects on lipid peroxidation (LP) of *M. melissophyllum* extracts have been evaluated using the Fe^2+^/ascorbate system of induction, by the TBA-assay (Figure 2). Inhibition of LP was determined by measuring the formation of secondary components (mainly MDA) of the oxidative stress, using liposomes as an oxidizable substrate. In general, all of the examined extracts (except n-BuOH extract) expressed strong antioxidant capacity. Protective activity can be explained by present of phenolic acids and their influence on antioxidative capacity of ascorbic acid, which doesn`t show a strong antioxidative effect in lipid phase, but different phenolic compounds can result increase of its antioxidant activity [23]. The largest inhibitory activity, again, was exhibited by water extract. The antioxidant activities of all extracts of *M. melissophyllum* leaves were dose dependent.

The *n*-BuOH extract shows a prooxidative effect that is increased by increasing concentration of added extract. It can be supposed that compounds with polar groups were extracted by *n*-BuOH, and are present in high concentration in the extract. It is notable that molecules which show antioxidant activity, when they are present in high concentration, might behave as prooxidants [24], so *n*-BuOHextract of *Melittis melissophyllum* L. leaves probably have this kind of activity. 

### 2.2. In vivo experiments

The represented antioxidant activity results show that extracts of examined plant species, specially H_2_O extract, are efficient in the protection of tissues and cells from oxidative stress. Anyway, according to variations in regard to antoxidant activity of tested by different *in vitro* models, there are also requiste *in vivo* tests that would confirm antioxidant activity. *In vivo* tests are also necessary because a lot of plant phenols are biotransformed during their active metabolism. The experimental animals were given 1 mL/kg of 2% of Et_2_O, CHCl_3_, EtOAc, *n*-BuOH or H_2_O extract (i.p.) of *M. melissophyllum* leaves for 7 days. After 7 days, the animals were sacrificed. In the liver homogenate and blood-hemolysate of sacrificed animals the following biochemical parameters were determined: LPx intensity, content of GSH and activities of GSHR, GSHPx, Px, XOD and CAT (Table 3 and Table 5). In Table 4 and Table 6 the results of the same parameters obtained after pretreatment of experimental animals with the examined *M. melissophyllum* extracts, followed by a single dose of carbon tetrachloride (CCl_4_) as a well-known radical generator are presented.

As can be seen from Table 3. the EtOAc extract decreased the GSHPx activity; Et_2_O extract caused its increase, while the other three extracts caused no essential changes of this parameter. The EtOAc extract decreased the GSH content compared with control. All permanent extracts increased the index of GSH, but only the increase caused by treatment with H_2_O extract are statistically remarkable. Since about 95% of glutathione in liver is reduced and only 5% is oxidized [25], this increase in the GSH index by applying by *M.* m*elissophyllum* L. leaves extracts (except EtOAc extract) probably derives from some compounds that contains a free SH-group, regardless if these compounds are present in extract or secondary biomolecules from these extracts have influenced an increase in the synthesis of GSH or cysteine. Treatment with the Et_2_O, CHCl_3_, and H_2_O extracts yielded an increase in GSHR activity, whereas the EtOAc extract caused a statistically significant decrease of this enzyme, which was in agreement with the action of this extract on GSH. 

**Table 3:** Effect of extracts of *M. melissophyllum* leaves on the biochemical parameters in the liver homogenate.

Parameter	Control	Extract				
		Et_2_O	CHCl_3_	EtOAc	*n-*BuOH	H_2_O
GSHPx	3.43 ± 0.17	3.96 ± 0.16^a^	3.32 ± 0.18	2.84 ± 0.21^a^	3.37 ± 0.15	3.46 ± 0.18
GSH	2.61 ± 0.13	2.76 ± 0.11	2.86 ± 0.14	2.12 ± 0.17^a^	2.87 ± 0.22	3.49 ± 0.13^a^
GSHR	3.96 ± 0.18	4.82 ± 0.21^a^	5.36 ± 0.25^a^	2.98 ± 0.14^ a^	3.96 ± 0.19	5.52 ± 0.23^a^
Px	4.38 ± 0.13	4.81 ± 0.17^ a^	4.93 ± 0.21^ a^	3.89 ± 0.13^ a^	5.11 ± 0.25^ a^	4.87 ± 0.15^ a^
LPx	7.19 ± 0.23	7.36 ± 0.21	7.91 ± 0.19^a^	6.71 ± 0.16^ a^	7.12 ± 0.23	6.19 ± 0.27^ a^
CAT	4.41 ± 0.16	3.83 ± 0.17^ a^	5.03± 0.19^a^	3.20 ± 0.15^ a^	4.52 ± 0.11	5.49 ± 0.13^a^
XOD	5.27 ± 0.17	6.17 ± 0.23^a^	6.02 ± 0.16^a^	4.23 ± 0.16^ a^	5.11 ± 0.22	4.17 ± 0.19^ a^

T-test ^a^ p ≤ 0.05 n = 5; x ± SD. Content of GSH is expressed in nmol GSH/mg of protein. Activities of GSHPx, GSHR, Px, CAT and XOD are expressed in nmol/mg of protein x min^−1^. Intensity of LPx is expressed in nmol malondialdehyde/mg of protein.

On the other hand, all the extracts (except the EtOAc extract) produced a statistically significant increase in Px activity. In addition to the very important role of peroxidase in the oxidative stress there are literature data on some other actions of peroxidases. Thus, some plant peroxidases oxidize phenols to phenoxy radicals to form polymers and enable their removal from industrial wastewaters [26]. Having in mind the results presented in Table 1 and Figure 1 it might be interesting to test the aqueous extract of *M. melissophyllum* as a biological marker. As compared with control, intensity of LPx is statistically saignificant reduced during the treatment with ethylacetate and water extracts of * M. melissophyllum* leaves. The result derived by treatment with ethylacetate and water extracts is in according with amounts got *in vitro* experiment (Figure 2). Using CHCl_3_ extract leads to a significant increase of LPx intensity, whereas the other two extracts had no effect on this parameter. The CAT increased in the treatments with CHCl_3_ and H_2_O extracts, and decreased in the treatment with the Et_2_O and EtOAc extract. *n*-BuOH extract caused no essential changes of CAT with respect to control. An increased XOD value was observed only in the treatments with the Et_2_O and CHCl_3_ extracts. 

Table 4 presents the values of biochemical parameters obtained for the liver homogenate of animals treated with extracts of *M. melissophyllum* leaves and CCl_4_. In comparison with the control (animals received only physiological solution), treatment with CCl_4_ yielded a significant decrease in activities of all the enzymes (GSHPx, GSHR, Px, CAT, XOD), as well as of GSH content, the only increase being in the intensity of LPx. In combination with CCl_4_ the extracts exhibited different effects on GSHPx: while the CHCl_3_ and H_2_O extracts showed a statistically significant increase, the EtOAc extract decreased the activity of this enzyme. The Et_2_O and *n*-BuOH extracts in combination with CCl_4_ had no effect on the GSHPx activity. 

**Table 4:** Effect of *M. melissophyllum* leaves extracts and CCl_4 _on the liver homogenate biochemical parameters.

Parameter	Control + CCl_4_	Extract + CCl_4_				
		Et_2_O	CHCl_3_	EtOAc	*n*-BuOH	H_2_O
GSHPx	2.12 ± 0.17	2.31 ± 0.15	2.61 ± 0.18^a^	1.62 ± 0.23^a^	2.18 ± 0.24	2.58 ± 0.18^a^
GSH	2.26 ± 0.17	2.18 ± 0.12	2.35 ± 0.19	1.86 ± 0.12^ a^	1.92 ± 0.15^ a^	2.49 ± 0.25
GSHR	2.53 ± 0.21	3.46 ± 0.28^a^	4.25 ± 0.24^a^	2.08 ± 0.15^a^	2.26 ± 0.26	3.07 ± 0.26^a^
Px	3.47 ± 0.18	3.05 ± 0.17^a^	2.97 ± 0.25^a^	2.84 ± 0.21^a^	3.06 ± 0.23	3.01 ± 0.24^a^
LPx	8.91 ± 0.29	7.12 ± 0.21^a^	7.06 ± 0.24^ a^	6.92 ± 0.17^a^	6.98 ± 0.24^ a^	6.81 ± 0.24^a^
CAT	2.08 ± 0.17	2.17 ± 0.22	2.47 ± 0.25	1.02 ± 0.12^a^	2.10 ± 0.14	1.44 ± 0.18^a^
XOD	4.61 ± 0.25	3.69 ± 0.23^a^	5.38 ± 0.21^a ^	3.02 ± 0.19^a^	3.98 ± 0.17^ a^	2.39 ± 0.14^ a^

T-test ^a^ p ≤ 0.05 n = 5; x ± SD. Content of GSH is expressed in nmol GSH/mg of protein. Activities of GSHPx, GSHR, Px, CAT and XOD are expressed in nmol/mg of protein x min^−1^. Intensity of LPx is expressed in nmol malondialdehyde/mg of protein.

Treatment with CCl_4_ caused no significant reduction of GSH content compared with that seen in untreated animals. Combined treatment with the extracts and CCl_4_ had a different effect on the GSH content in the liver homogenate. While the EtOAc and *n*-BuOH extracts caused a decrease, the Et_2_O, CHCl_3_ and H_2_O extracts showed no effect on this parameter. The Et_2_O, CHCl_3_ and H_2_O extracts in combination with CCl_4_ increased the GSHR activity in the liver homogenate and the EtOAc extract reduced it. On the other hand, the *n*-BuOH extract did not influence this parameter. Bearing in mind the fact that the CCl_4_-induced oxidative injuries of the liver require a high consumption of GSH, which is regenerated via the GSHR activity, it can be concluded that the extracts showed no protective effect, as they did not cancel out the effect of treatment with CCl_4_. All the extracts (except *n*-BuOH one) in combination with CCl_4_ yielded a statistically significant decrease in Px, the EtOAc extract exhibiting the strongest effect. All extracts of *M. melissophyllum* leaves combine with CCl_4_ have showed a statistically significant decrease of LPx intensity, and this behavior of the extract probably results from the presence of secondary biomolecules like flavonoids and phenolic acids. Handa *et al.* [27] determined that secondary biomolecules such as flavonoids, xanthones and tannins in combination with CCl_4_ have protective effects on liver. Phenolic components present in *M. melissophyllum* leaves (rutin, luteolin, kvercetin) are known as strong inhibitors of CCl_4_-induced LP [28]. Flavonoids could affect the initiation phase of lipid peroxidation, where they influence the metabolism of CCl_4, _they scavenge the free radicals, or they decrease the microsomal enzyme systems that are claimed for CCl_4_ metabolism [29]. In continuation of this process, flavonoids can scavenge lipoperoxides and their radicals or they can act as chelating agents for Fe^2+^ ion, and in this way can stop Fenton reactions [30]. On the basis of these results, we can conclude that all of extracts of *M. melissophyllum* leaves showed protection effect in relation to the CCl_4_-induced lipid peroxidation. The EtOAc and H_2_O extracts in combination with CCl_4_ yielded a statistically significant decrease in CAT activity. Administration of CCl_4_ significantly decreased the activity of XOD (4.61 ± 0.25 nmol/mg of protein x min^-1^) compared with the untreated animals (5.27 ± 0.17 nmol/mg of protein x min^-1^). Further, the Et_2_O, EtOAc, *n*-BuOH and H_2_O extracts in combination with CCl_4_ significantly lowered the activity of XOD. On the contrary, CHCl_3_ extract significantly increased XOD activity. Some recent studies point to the relationship between elevated XOD activity and oxidative stress in hypertension and the production of oxygen radicals in diabetes [31]. However, allopurinol, a XOD inhibitor known in clinical practice, reduces oxidative stress in diabetes [32], interacting with some peroxy radical species, such as Cl_3_OO^•^. It can be supposed that the active constituents present in Et_2_O, EtOAc, *n*-BuOH and H_2_O extracts act similarly, reducing the activity of this enzyme.

Like those given in Table 3, the results in Table 5 show that all the extracts caused a statistically significant decrease in blood GSHPx activity. All extracts reduced the GSH content in the hemolysate too, the decrease being statistically significant. The activity of GSHR decreased after treatment with Et_2_O, EtOAc and *n*-BuOH extracts. 

**Table 5:** Effect of extracts of *M. melissophyllum* leaves on the biochemical parameters in blood hemolysate.

Parameter	Control	Extract				
		Et_2_O	CHCl_3_	EtOAc	*n*-BuOH	H_2_O
GSHPx	5.94 ± 0.22	4.81 ± 0.16^a^	4.95 ± 0.19^a^	2.77 ± 0.23^a^	4.79 ± 0.16^a^	3.89 ± 0.16^a^
GSH	6.78 ± 0.12	6.22 ± 0.14^a^	6.13 ± 0.17^a^	5.56 ± 0.21^a^	3.96 ± 0.21^a^	4.37 ± 0.19^ a^
GSHR	7.67 ± 0.24	6.17 ± 0.24^a^	7.87 ± 0.27	5.56 ± 0.17^a^	6.81 ± 0.19^a^	7.72 ± 0.28
Px	3.72 ± 0.17	3.39 ± 0.21	3.69 ± 0.25	2.94 ± 0.19^a^	3.46 ± 0.27	3.65 ± 0.18
LPx	4.81 ± 0.24	4.59 ± 0.28	3.78 ± 0.17^a^	2.96 ± 0.13^a^	4.74 ± 0.19	4.07 ± 0.24^a^
CAT	4.28 ± 0.26	4.77 ± 0.18^a^	3.75 ± 0.19^a^	3.18 ± 0.16^a^	4.11 ± 0.18	3.86 ± 0.23
XOD	4.76 ± 0.29	5.92 ± 0.31^a^	5.44 ± 0.26^a^	5.58 ± 0.19^a^	5.41 ± 0.27^a^	5.48 ± 0.24^a^

T-test ^a^ p ≤ 0.05 n = 5; x ± SD. Content of GSH is expressed in μmol GSH/mL erythrocytes; Activities of GSHPx, GSHR, Px, CAT and XOD are expressed in nmol/mL erythrocytes x min^−1^; Intensity of LPx is expressed in nmol malondialdehyde/mL erythrocytes.

As for the Px activity, all extracts reduced the activity of this enzyme, the difference being statistically significant only with the EtOAc extract. Three extracts, CHCl_3_, EtOAc and H_2_O, induced a significant decrease of LPx intensity, while the Et_2_O and *n*-BuOH ones decreased the level of this enzyme insignificantly. A statistically significant decrease in CAT activity was produced by the CHCl_3_ and EtOAc extracts. On the other hand, all extracts caused a statistically significant increase in XOD activity. XOD is an enzyme which is present in measurable amounts only in liver and jejunum. Otherwise, because of different irregularities in liver, this enzyme should be cleared by the circulation. Consequently, an increase of XOD in the blood could be an indicator of liver damage [33]. Nowadays, increase of XOD activity is related to amplification of oxidative stress and production of free radicals [34]. From the results obtained during this research, we can conclude that the high level of XOD in the experimental animals blood-hemolysate that were treated by *M. melissophyllum* leave extracts is probably consequent of liver damage, so these extracts showed hepatotoxic effects. High production of oxygen radicals (priority O_2_^−^^·^) and H_2_O_2_ probably influenced the increased production of XOD. We must bear in mind that only one of two forms of XOD is related to high production of oxygen radicals, while the other form has a completely different metabolic way, a mechanism proposed by Kisher *et al.* [35]. According to this mechanism, results obtained from this research could be interpreted otherwise. Particulary, the other form of XOD leads to the formation of NADH+H^+^, which could be included in the respiratory chain with a view to synthesize ATP, that could be interpreted as the ultimate eventual protective effect of *M. melissophyllum* leave extracts.

In Table 6 the results of biochemical parameters measured in the blood-hemolysate of animals treated with extracts of *M. melissophyllum* leaves and CCl_4_ are presented. The activity of glutathione peroxidase (GSHPx) in the blood of animals treated with CCl_4_ (Table 6) was increased compared with the control (Table 5). However, only the administration of EtOAc and *n*-BuOH extracts significantly decreased the activity of GSHPx. Other administered extracts did not cause notable changes. 

**Table 6:** Effect of extracts of *M. melissophyllum* leaves and CCl_4_ on the biochemical parameters in blood hemolysate.

Parameter	Control + CCl_4_	Extract + CCl_4_				
		Et_2_O	CHCl_3_	EtOAc	*n*-BuOH	H_2_O
GSHPx	6.08 ± 0.17	5.83 ± 0.21	5.77 ± 0.24	4.69 ± 0.21^a^	4.94 ± 0.27^a^	5.91 ± 0.33
GSH	5.21 ± 0.13	4.84 ± 0.19^a^	3.97 ± 0.24^a^	4.08 ± 0.17^a^	3.03 ± 0.18^a^	3.86 ± 0.24^a^
GSHR	6.12 ± 0.29	5.76 ± 0.28	5.87 ± 0.22	4.12 ± 0.18^a^	6.17 ± 0.29	6.58 ± 0.32
Px	4.08 ± 0.22	3.27 ± 0.16^a^	4.86 ± 0.24^a^	3.15 ± 0.23^ a^	3.04 ± 0.18^ a^	4.11 ± 0.29
LPx	5.11 ± 0.24	5.31 ± 0.17	4.92 ± 0.21	3.02 ± 0.24^a^	5.17 ± 0.25	2.98 ± 0.12^a^
CAT	3.74 ± 0.24	3.19 ± 0.23^a^	3.24 ± 0.19^a^	3.07 ± 0.25^a^	2.98 ± 0.18^a^	3.13 ± 0.28^a^
XOD	6.19 ± 0.31	6.08 ± 0.26	4.96 ± 0.24^a^	5.11 ± 0.17^a^	6.97 ± 0.25^a^	5.23 ± 0.21^a^

T-test ^a^ p ≤ 0.05 n = 5; x ± SD. Content of GSH is expressed in μmol GSH/mL erythrocytes; Activities of GSHPx, GSHR, Px, CAT and XOD are expressed in nmol/mL erythrocytes x min^−1^; Intensity of LPx is expressed in nmol malondialdehyde/mL erythrocytes.

The amount of GSH from the blood of non-CCl_4_ treated animals was compered with CCl_4_ treated animals (5.21 ± 0.13 *vs*. 6.78 ± 0.12), and it was observed that the amount of GSH is one of the important secondary biomolecules, that take a part in different detoxification processes in organism. Not only “radical“ GSH already in conjugation with toxic metabolites protects organism. From the results presented it is obvious that CCl_4_ decreased the values of GSHR (6.12 ± 0.29 nmol/mL erythrocytes x min^−1^), compared with the levels in the control group (7.67 ± 0.24 nmol/mL erythrocytes x min^−1^). The results of GSHR activity were significantly lower only in the combination of CCl_4 _with EtOAc extract (4.12 ± 0.18 nmol/mL erythrocytes x min^−1^). Also, treatment with Et_2_O and CHCl_3_ extracts caused a decrease of GSHR activity, but not notably. Treatment with CCl_4_, compared with the control animals (Table 5 and Table 6) did not cause notable differences in the activity of Px. Application of three extracts (Et_2_O, EtOAc or *n*-BuOH) with CCl_4_, caused a statistically significant decrease of Px. On the other hand, the CHCl_3_ extract significantly increased the activity of Px in combination with CCl_4_. Similar to the values of Px, values of LPx showed an insignificant increase of activity in the blood of animals treated with CCl_4_ (Table 6), compared with the control (Table 5). A clear ‘protective’ effect was seen in experimental animals administered H_2_O extract and CCl_4_ compared with untreated animals. Furthermore, EtOAc extract also significantly decreased the activity of LPx, while Et_2_O, CHCl_3_ and *n*-BuOH extracts did not change notably the levels of lipid peroxidation. In all previous research works (Figure 2, Table 3, Table 4, Table 5), H_2_O extract showed the best protective effect on LPx. Already it was noticed that these protective properties probably come from secondrary biomolecules such as the flavonoids and phenolic acids are. Administration of CCl_4_ caused a decrease in the values of CAT activity in blood of experimental animals compared with the control. Combined treatment of experimental animals with CCl_4_ and all of examined extracts reduced the activity of this enzyme, especially in the case of the *n*-BuOH extract. The level of activity of xanthine oxidase (XOD) was increased in the blood of experimental animals after administration of CCl_4_ (Table 6) with respect to the untreated ones (Table 5). On the contrary, lower activities of XOD were obtained after combined treatment of CCl_4_ with CHCl_3_, EtOAc or H_2_O extracts. However, only the *n*-BuOH extract exhibited a significant increase of XOD activity. 

## 3. Experimental

### 3.1. General

Leaves of cultivated *Melittis melissophyllum* L., Lamiaceae were collected in May 2008 in the city of Vrdnik, Republic of Serbia. A voucher specimen of the collected leaves (*Melittis melissophyllum* L. 1753 subsp. *melissophyllum*, No 2-1810, Serbia, Fruška Gora, Vrdnik (UTM 34T DQ 1 09), 17.05.2008., det.: Goran Anačkov) was confirmed and deposited at the Herbarium of the Department of Biology and Ecology (BUNS Herbarium), Faculty of Natural Sciences, University of Novi Sad.

The plant leaves were dried in air and ground in a mixer. An amount of 200 g of the finely powdered material was extracted three times with 4 L of 70% methanol (MeOH) during a 24-h period. After removal of MeOH under reduced pressure, the aqueous phase was successively extracted with four solvents of increasing polarity, namely ether (Et_2_O), chloroform (CHCl_3_), ethylacetate (EtOAc) and *n*-butanol (*n*-BuOH). The extraction was carried out until a colourless extract was obtained. The residue was the aqueous extract. All of five extracts (Et_2_O, CHCl_3_, EtOAc, *n*-BuOH, and H_2_O) were evaporated to dryness and then dissolved in 50% ethanol to make 10% (w/v) solutions. These solutions, either as such or in diluted state, were used in the subsequent experiments.

### 3.2. In vitro experiments

Determination of total flavonoids was conducted by a colorimetric method based on the property of flavonoids and flavon glycosides to build metal complexes with aluminum ions [36]. Absorbance of analyzed solutions is measured at λ = 430 nm.

The DPPH assay was performed as described before [37], following the transformation of the DPPH radical to its reduced, neutral form (DPPH-H). The samples of all extracts of *M. melissophyllum* leaves were investigated in concentrations of 2.50–25.00 μg/mL. The same procedure was repeated with *tert*-butylated hydroxytoluene (BHT) as a positive control. For each sample five replicates were recorded. The disappearance of DPPH was measured spectrophotometrically at 515 nm. 

Superoxide anion radicals were generated in the system xanthine/xanthine-oxidase, and the quantity of O_2_^·^^−^ was determined by the nitrite method [38] with modifications. The samples of all extracts of *M. melissophyllum* leaves were investigated in concentrations of 2.50–25.00 μg/mL. The same procedure was repeated with BHT as a positive control. For each sample five replicates were recorded. The intensity of color was measured spectrophotometrically (λ = 550 nm).

Production of NO radicals was determined spectrophotometrically. NO radical generated from sodium-nitropruside (SNP) reacts with oxygen in water solution at a physiological pH to give nitrite ions. Concentration of nitrite anions was determined using Griess reagent [39,40]. At room temperature nitrite ions react with Griess reagent and form purple complex. The samples of all extracts of *M. melissophyllum* leaves were investigated in concentrations of 2.50–25.00 μg/mL. The intensity of color, which is the function of the nitrite concentrations, was measured spectrophotometrically (λ = 546 nm). For each sample, five replicates were recorded. 

The percentage of RSC for each radical was calculated using the following equation:

RSC(%) = 100 × (*A*_blank_-*A*_sample_ ⁄ *A*_blank_)

From the obtained RSC values, the IC_50_ values, which represented the concentrations of the essential oils that caused 50% neutralization, were determined by linear regression analysis.

Scavenging capacity of the *M. melissophyllum* leaves extracts for hydroxyl radicals was determined by monitoring the chemical degradation of 2-deoxy-D-ribose [41]. The reaction was initiated by hydroxyl radicals obtained in Fenton’s reaction [3], which yields products that react with thiobarbituric acid (TBA test). The obtained products, among which malondialdehyde (MDA) is the most important, are determined by a spectrophotometric method at 532 nm. The absorbance of the resulting solutions and the blank (with same chemicals, except sample) was recorded. For the experiment, three concentrations of extracts of bastard leaves were prepared (10%, 5% and 1% solution). Five replicates were performed for each sample. 

The absorbance read at the end of the experiment was used for the calculation of the percentage inhibition of deoxyribose degradation by the examined extracts:

*I*(%) = 100 × (*A*_blank_-*A*_sample_ ⁄ *A*_blank_)

The extent of LP was determined by measuring the color of the adduct produced in the reaction between 2-thiobarbituric acid (TBA) and malondialdehyde (MDA), as an oxidation product in the peroxidation of membrane lipids, by the TBA assay [3,42,43]. The commercial preparation of liposomes ‘PRO-LIPO S’ (Lucas-Meyer) pH = 5–7 was used as a model system of biological membranes. The liposomes, 225–250 nm in diameter, were obtained by dissolving the commercial preparation in demineralized water (1:10), in an ultrasonic bath. For the experiment, three concentrations of extracts of *M. melissophyllum* leaves were prepared (10%, 5% and 1% solution). Five replicates were performed for each sample.

The percentage of LP inhibition was calculated by the following equation:

*I*(%) = (*A*_0_ – *A*_1_) ⁄ *A*_0_ × 100

where *A*_0_ was the absorbance of the control reaction (full reaction, without the test compound) and *A*_1_ was the absorbance in the presence of the inhibitor.

### 3.3. *In vivo* antioxidant activity

This investigation was conducted on sexually mature white laboratory mice (both sexes), type BALB/C, with an average body weight of 20–25 grams. Animal care and all experimental procedures were conducted in accordance with the *Guide for the Care and Use of Laboratory Animal Resources*, edited by Commission of Life Sciences, National Research Council (USA). Experimental animals were bred in the vivarium at the Department of Pharmacology, Toxicology and Clinical Pharmacology, Medical Faculty, University of Novi Sad, Serbia. Animals were kept in standard plexiglass cages (room temperature 21 ± 1 °C; humidity 55 ± 1.5%, with 12 h light period). They were fed standard laboratory animals feed, produced by the Veterinary Institute in Zemun. Animals were given free access to food and fluid (water). Animals were randomly assigned into six groups, consisting of 10 animals each. The animals of control group were given intraperitoneally (i.p.) physiological solution, the animals of 5 experimental groups received at the same time (i.p., 1.0 mL/kg) one of five *M. melissophyllum *leaves extracts. After 7 days five animals from each group were sacrificed to determine the biochemical liver and blood parameters. The remaining five animals of each group were treated (i.p.) with CCl_4 _in olive oil (1:1, 2.0 mL/kg) and sacrificed 24 h later, to determine the same biochemical parameters.

Examined biochemical parameters were measured in blood hemolysate and liver homogenate. Liver was homogenized in a Potter homogenizer with TRIS-HCl/sucrose in a ratio of 1:3 at 4 °C. Obtained homogenate was filtered. The following biochemical parameters were analyzed in blood hemolysate and liver homogenate: extent of lipid peroxidation (LPx) was determined after Buege and Aust [43], peroxidase (Px) activity was measured after Simon *et al*. [44], catalase activity (CAT) after Beers and Sizer [45]. Glutathione peroxidase (GSHPx) activity was evaluated as described by Chin *et al*. [46], glutathione reductase (GSHR) after Glatzle *et al. * [47], xanthine oxidase (XOD) after Bergmayer [48], content of reduced glutathione (GSH) after Kapetanović and Mieyal [49]. The total protein content in liver was determined after Gornall *et al*. [50].

### 3.4. Chemicals

Thiobarbituric acid (TBA), xanthine, xanthine-oxidase, ethylenediaminetetraacetic acid (EDTA), 2,2-diphenyl-1-picrylhydrazil (DPPH) were obtained from Sigma Chemicals (St. Louis, MO, USA). 2-Deoxy-D-ribose was purchased from Aldrich. *N***-**(1-Naphthyl)-ethylenediamine dihydrochloride (NEDA), *n*-hexane, DTNB, and reduced glutathione were obtained from Merck (Darmstadt, Germany). *tert*-butylated hydroxytoluene was obtained from Fluka, AG (Buchs, Switzerland). The commercial preparation of liposomes “PRO-LIPO S” was purchased from Lucas-Meyer (Hamburg, Germany). All chemicals used were of analytical grade.

### 3.5. Statistical analysis

Results of biochemical analyses are presented as mean value ± standard deviation (S.D.). The differences between control and test groups were analyzed using the Student t-test (significant difference at p ≤ 0.05 confidence level).

## 4. Conclusions

In conclusion, the results of the antioxidant effects of the investigated extracts of *M. melissophyllum*, obtained with different methods of assessment, point out very strong protective activities, both as free radical scavengers and LP inhibitors. In particular, the H_2_O extract showed the strongest effect in the neutralization of free radicals, as the IC_50_ value was achieved at the lowest concentration. Also, a very strong protective activity of the EtOAc and H_2_O extracts in lipid peroxidation processes was recorded, which means that they may have a protective role in oxidative stress. Based on the experimental results, the strongest protective effect in *in vitro* experiments was shown by the H_2_O extract. Furthermore, *M. melissophyllum* leaves extracts exhibited different activities in relation to the investigated biochemical parameters. The results obtained indicate toxicity of CCl_4_, probably due to the radicals involved in its metabolism. Although the *in vitro* testing, the H_2_O extract showed the best protective properties, in *in vivo* experiments the best protective effect was shown by the EtOAc extract. 
